# Mapping Data to Deep Understanding: Making the Most of the Deluge of SARS-CoV-2 Genome Sequences

**DOI:** 10.1128/msystems.00035-22

**Published:** 2022-03-21

**Authors:** Bahrad A. Sokhansanj, Gail L. Rosen

**Affiliations:** a Drexel University, Ecological and Evolutionary Signal-Processing and Informatics Laboratory, Department of Electrical & Computer Engineering, College of Engineering, Philadelphia, Pennsylvania, USA; Tufts University

**Keywords:** COVID-19, SARS-CoV-2, viral genomics, bioinformatics, deep learning, explainable AI, genomics, machine learning

## Abstract

Next-generation sequencing has been essential to the global response to the COVID-19 pandemic. As of January 2022, nearly 7 million severe acute respiratory syndrome coronavirus 2 (SARS-CoV-2) sequences are available to researchers in public databases. Sequence databases are an abundant resource from which to extract biologically relevant and clinically actionable information. As the pandemic has gone on, SARS-CoV-2 has rapidly evolved, involving complex genomic changes that challenge current approaches to classifying SARS-CoV-2 variants. Deep sequence learning could be a potentially powerful way to build complex sequence-to-phenotype models. Unfortunately, while they can be predictive, deep learning typically produces “black box” models that cannot directly provide biological and clinical insight. Researchers should therefore consider implementing emerging methods for visualizing and interpreting deep sequence models. Finally, researchers should address important data limitations, including (i) global sequencing disparities, (ii) insufficient sequence metadata, and (iii) screening artifacts due to poor sequence quality control.

## PERSPECTIVE

COVID-19 has been called the “first pandemic in the post-genomic era” (1). The first severe acute respiratory syndrome coronavirus 2 (SARS-CoV-2) genome was published on 12 January 2020, a week after the WHO first reported on the virus. Only 5 days later, the sequence was used to design the mRNA vaccines that have changed the course of the pandemic (2). Since then, next-generation sequencing technology has enabled an unprecedented view of genetic changes in the virus throughout both the duration of the pandemic and different parts of the world (1, 3). Global data sharing of sequence data has been equally critical, much to the credit of the GISAID EpiCoV database project (4). GISAID’s primary mission has been to share flu genomes, in part to help design the annual flu vaccine (its full name being the Global Initiative on Sharing All Influenza Data) (5). Now, at the beginning of 2022, the GISAID EpiCoV database has accumulated nearly 7 million SARS-CoV-2 genome sequences, and at present, around 800,000 sequences are being added each month. So much data has been generated and made available that it has spurred the development of computational tools for high-frequency sequence variant tracking (6) and even daily updates (7, 8).

Despite the surge in research efforts devoted to COVID-19 (9, 10), laboratory study of the virus remains a more specialized and time-consuming effort than sequencing. Clinical and epidemiological data are often superficial, measuring only a few variables, other than data sets specific to particular facilities or narrow populations. We need to fully capitalize on the abundant data that we do have to (i) anticipate how changes in the virus might affect health before we have time to gather empirical data and (ii) better design and interpret experiments to maximize our use of limited resources. So, how can we translate genome sequence data to as much biological understanding and actionable clinical insight as possible?

## SARS-COV-2 IS RAPIDLY EVOLVING

SARS-CoV-2 has spent the first 2 years of the pandemic rapidly evolving in ways that have had a big impact on virulence, transmission, and ability to evade our immune responses (11). SARS-CoV-2 is an RNA virus, so its genome is prone to mutate—albeit at a rate mitigated by its large genome size and the proofreading function of its exoribonuclease (12). The most frequent mutations observed in coronaviruses are generally substitutions, although insertions and deletions are observed as well (13). In some cases, insertions from other viral genomes may occur, and, in fact, it appears as though the SARS-CoV-2 genome includes an insertion from human RNA (14). In other human coronaviruses, the estimated mutation rate is around 3 × 10^−4^ substitutions per site per year (15, 16).

The amount of mutation observed during the COVID-19 pandemic has been even more substantial than expected (17). An early estimate of SARS-CoV-2 mutation was 6 × 10^−4^ substitutions per site per year (18). But the disease has spread widely around the world since then, and novel variants transmit more quickly—increasing the opportunities for the virus to mutate (19, 20). The SARS-CoV-2 spike protein will continue to change in the future. Studies on another human coronavirus, HCoV-OC43, suggest that genetic drift plays a role in coronavirus adaptive evolution (21). One study estimates that as of July 2021, SARS-CoV-2 had only “explored” 31% of the potential space for spike gene variation, based on comparisons with related sarbecoviruses (22).

## SEQUENCE ANALYSIS HAS STRUGGLED TO KEEP UP

The first widely used tool for tracking SARS-CoV-2 genomic variation was the Nextstrain project, https://nextstrain.org. Nextstrain, originally developed as a general tool for viruses, was adapted to offer clade definitions for SARS-CoV-2 based on phylogenetic analysis (23). Phylogenetic tree reconstruction has been effective in inferring viral origins and trace transmission changes but not as useful in classifying genomes because the virus can accumulate and drop mutations in parallel across clades and subclades (24). The Pango nomenclature (https://cov-lineages.org/), developed specifically for SARS-CoV-2, has largely supplanted Nextstrain clade definitions (25). New sequences are assigned to Pango classifications, called “lineages,” using the Random Forests classification algorithm. A new Pango lineage is defined when a sufficient number of viral sequences emerges with a phylogenetic dissimilarity from existing sequences above a set threshold (26). Particularly significant Pango lineages have been identified by the World Health Organization (WHO) as variants of concern (VOC), which are given Greek letter designations (27), such as Alpha (Pango lineage B.1.1.7), Beta (B.1.351), Delta (B.1.167.2), and, recently, Omicron (B.1.1.529).

While Pango lineages appear clear and well-defined, the reality is that the genome is much more fluid. If we want to understand how genome affects viral function, we cannot rely on traditional taxonomic categorization. As mutations recur, revert, and proliferate, taxonomy hits its limits of utility (11). As an initial matter, changes to SARS-CoV-2 properties often implicate combinations of multiple mutations that emerge simultaneously—and then sometimes revert in whole or in part as the virus continues to evolve (28, 29). For example, one frequent spike protein amino acid substitution, N501Y, has appeared and reverted contemporaneously in multiple clades and lineages, with no evidence of recombination (30). Simultaneous mutations can also have unpredictable, nonlinear effects, i.e., they can be synergistic, antagonistic, or fully independent (31). This complicates classical and Bayesian logistic regression methods for predicting fitness or protein function from mutations, as they rely on assuming the independence between mutations of individual amino acids or bases (32).

SARS-CoV-2 evolution is also highly nonlinear. Widespread lineages, such as Delta, have spawned complex sublineages with distinct immune evasion and virulence properties, which often genetically share more in common with distantly related lineages than their most recent ancestor (33, 34). The increasingly complex evolutionary history of the virus stymies other proposed methods for genetically subtyping viral variants as well (35–37). Further complicating the picture, some immunocompromised individuals can have chronic infections lasting 6 months to a year (38). During long-term infection, a spike protein can emerge with multiple variations, which phylogenetic analysis identifies as “long branch” divergence from the phylogenetic tree (39). Some long-term patients may even be treated with convalescent plasma or antibodies, which may select for immune evasive mutations (40). The Omicron variant has such a long branch divergence, indicating that it may have emerged in an immunocompromised host or after incubating in a nonhuman host such as mice (41, 42).

## CAN DEEP SEQUENCE LEARNING HELP?

How can we predict the virulence, fitness, antibody evasion, and other key properties of novel SARS-CoV-2 variants from complex, nonlinear changes in genetic sequence? Machine learning can tackle complex pattern recognition problems by training a model that can classify the organisms or genes by phylogeny or phenotype based on features of their genetic sequences. For example, we can extract *k*-mer (short subsequence) frequencies or other combinations of bases/amino acids and use them as features to train classifiers using naive Bayes classifier (NBC), support vector machines (SVM), decision tree-based methods, and neural networks (43–49). Machine learning with *k*-mer features has been used for SARS-CoV-2 to identify genetic fingerprints of specific infections (50), classify variants (51, 52), and train a model to predict the pathogenicity of unknown viruses (53). Another approach is to build profile hidden Markov models (HMMs), which can identify taxonomic lineages and variants of viruses. HMMs have been used to align SARS-CoV-2 sequences and compare its spike protein to that of other coronaviruses (22, 54, 55).

Deep learning has emerged as an even more powerful and flexible tool to find patterns in large and complicated data sets (56–59). Deep learning models use multiple layers of neural networks to automatically extract and transform features during training (56–58). We can borrow deep learning methods developed for natural language processing (NLP) to find patterns in sequence data, where bases and amino acids that make up genome and protein sequences are analogous to semantic relationships between the words that make up sentences (60–63). For example, one group of researchers has used concepts from semantic processing, e.g., the frequency of correlated words, to identify potential mutagenic sites in viruses including SARS-CoV-2 (64). An emerging approach to deep sequencing learning is to transform protein sequences to embeddings that reflect their semantic structure, using the BERT (bidirectional encoder representations from transformers) neural network architecture, which Google developed to handle natural language search (65–68). An example of this approach is *k*-means clustering of “ProtBERT” SARS-CoV-2 protein embeddings generated by pretraining a BERT model on millions of UniProt sequences, which can be used to identify mutational hot spots within the genome that may give rise to future variants (69).

A key goal for modeling is to predict the health risk of emerging variants before empirical data are available. To this end, our group has developed a deep learning model to predict patient outcomes for emerging sequence variants that takes into account patient demographics (70). Others are working to integrate sequence learning with computational protein structure models. For example, one project combines models of cell receptor binding and immune epitope alteration with transformer-based deep learning models to predict the fitness advantage of mutations (71). Deep learning has also been used to identify the relationship between protein sequence and function using data from deep mutational scanning, an experimental technique for massively parallel functional analysis of protein sequence site mutations (72, 73). Using this approach, another project predicts the risk for emerging variants by using a neural network to predict infectivity and vaccine breakthrough in combination with protein structure and binding prediction to model antibody resistance (74).

## LOOKING INSIDE THE DEEP LEARNING BLACK BOX

Deep learning methods excel at identifying complex features within data that allow classification. But they have a major weakness. Deep learning relies on neural networks, and it is very hard to determine why a neural network makes a particular classification or prediction. Interpretable, or explainable, machine learning can fill this important gap (75, 76). Interpretable machine learning is particularly important in bioinformatics, since explaining a model’s predictions is critical to justify making high-stakes clinical or research decisions based on machine learning predictions (77, 78). Accordingly, developers of deep learning approaches to SARS-CoV-2 should consider providing some functionality to interpret or explain predictions.

Analytical tools for interpretability in deep learning include examining neural network structure through relevance propagation, activation difference propagation, sensitivity analysis, and saliency map methods (79–81). Integrated gradients have been used to analyze RNA splicing models (82). An increasingly popular approach is the “attention” mechanism originally developed for NLP (83, 84). Attention can highlight important features in text processed by deep learning models (85–87). The amount of “attention” at a position in a sequence correlates with the weight put on that position in a trained model, where high attention at a position implies potential significance. Architectures combining convolutional neural networks (CNNs) with attention have been used to identify sequence motifs for functional genomics, e.g., transcription factor binding site detection (88, 89). Another group generated predictive models of adverse drug reactions based on chemical structures by combining attention with a CNN for each chemical property and structural feature in the model (90). Our group has shown that attention in combination with a recurrent neural network-based sequence model can provide insight into taxonomic and phenotypic classification of microbial 16s rRNA sequences (91), as well as gene ontology classifications of protein sequences (92).

Recently, transformer-based architectures have emerged, like the aforementioned BERT (93). Transformers are built on multiple attention modules (“heads”), which could be used for interpretability (94). For example, one recent paper demonstrated how different attention heads attended to different aspects of a learning task to identify nucleotide motifs for promoter sequences (95). However, attention cannot be inherently drawn out of transformers. Further processing steps are generally required to connect attention to specific linguistic features (96). Our group recently applied a self-attention layer after a transformer as a way to more readily extract and visualize attention across the sequence and applied it to SARS-CoV-2 (70). An important caveat is that, based on comparing attention to empirical evidence, attention does not necessarily imply explanation—at least in the sense of explaining precisely why a prediction took place (97). Attention can only highlight features that the attention layer of the deep learning model weighted most heavily during training, so it may only weakly indicate the complete set of important features for a classification problem.

## SEQUENCING DISPARITIES AND DATA CHALLENGES

**FIG 1:**
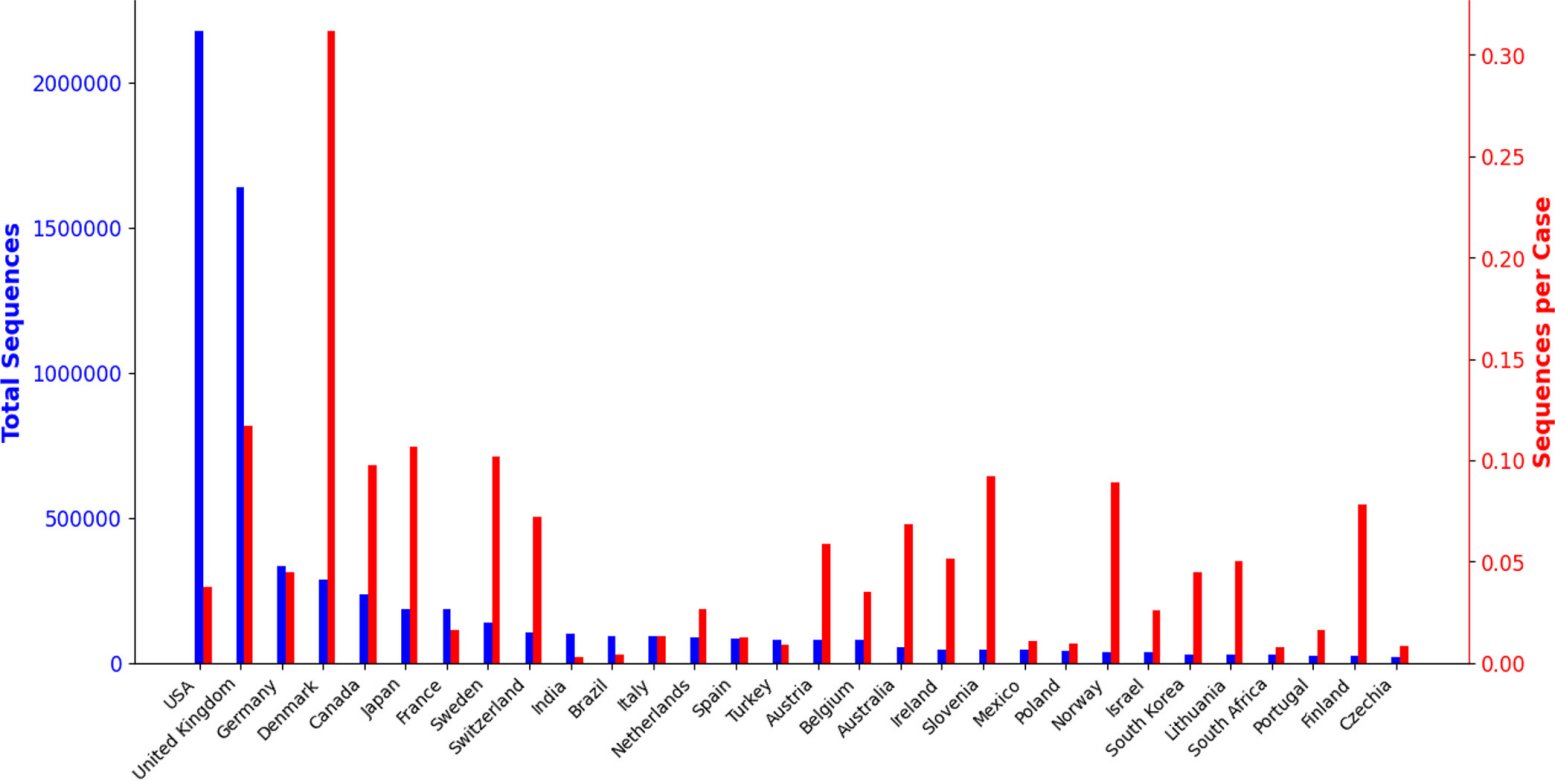
Total number of sequences submitted to GISAID (4) (left axis and bars, blue) and ratio of the number of submitted sequences to the total number of reported cases (104) as of 7 January 2022 for the 30 countries that have submitted the most sequences to GISAID. The overwhelming majority sequences in GISAID come from North America and Europe. Over half of all sequences are from the United States and United Kingdom alone. Sequencing rates show even greater disparities.

Finally, we highlight three important data limitations that researchers should address. First, as Fig. 1 shows, there are serious global inequities in sequencing data, with the overwhelming majority of sequences coming from Europe and North America. GISAID has encouraged data sharing from developing countries by trading restrictions on republishing sequence information for access to that information (98). But global sequencing resources are disparately available (99). Even within Europe and the United States, racial and regional disparities in sequencing found in other surveys (100) hamper SARS-CoV-2 sequencing as well. Second, the task of interpreting sequencing data is complicated by insufficient sample metadata, making it difficult to understand how SARS-CoV-2 sequences affect patient outcomes, for example. In GISAID, most sequences only have information about a patient’s age or gender (if available) and the location where the sample was collected. As of 7 January 2022, a little over 270,000 sequences (4%) of the nearly 6.9 million have any metadata for patient outcomes, and many metadata entries are unintelligible. Sequencing projects should be encouraged to collect and curate as much information as possible about the sample and meet minimum information standards for sequence metadata (101). Third, sequencing errors can lead to spurious results. Quality control is critical to make sure that low-frequency sequence variants are real (102). Sequences can pick up contaminants from other variants in the amplification process, leading to what appear to be recombinant variants but which are in fact simply artifacts (103).

## References

[1] van Dorp L, Houldcroft CJ, Richard D, Balloux F. 2021. COVID-19, the first pandemic in the post-genomic era. Curr Opin Virol 50:40–48. doi:10.1016/j.coviro.2021.07.002. 34352474 PMC8275481

[2] Corbett KS, Edwards DK, Leist SR, Abiona OM, Boyoglu-Barnum S, Gillespie RA, Himansu S, Schäfer A, Ziwawo CT, DiPiazza AT, Dinnon KH, Elbashir SM, Shaw CA, Woods A, Fritch EJ, Martinez DR, Bock KW, Minai M, Nagata BM, Hutchinson GB, Wu K, Henry C, Bahl K, Garcia-Dominguez D, Ma L, Renzi I, Kong WP, Schmidt SD, Wang L, Zhang Y, Phung E, Chang LA, Loomis RJ, Altaras NE, Narayanan E, Metkar M, Presnyak V, Liu C, Louder MK, Shi W, Leung K, Yang ES, West A, Gully KL, Stevens LJ, Wang N, Wrapp D, Doria-Rose NA, Stewart-Jones G, Bennett H, et al. 2020. SARS-CoV-2 mRNA vaccine design enabled by prototype pathogen preparedness. Nature 586:567–571. doi:10.1038/s41586-020-2622-0. 32756549 PMC7581537

[3] Chiara M, D'Erchia AM, Gissi C, Manzari C, Parisi A, Resta N, Zambelli F, Picardi E, Pavesi G, Horner DS, Pesole G. 2021. Next generation sequencing of SARS-CoV-2 genomes: challenges, applications and opportunities. Brief Bioinform 22:616–630. doi:10.1093/bib/bbaa297. 33279989 PMC7799330

[4] Khare S, Gurry C, Freitas L, Schultz MB, Bach G, Diallo A, Akite N, Ho J, Lee RT, Yeo W, Team GCC, Maurer-Stroh S. 2021. GISAID’s role in pandemic response. China CDC Wkly 3:1049–1051. doi:10.46234/ccdcw2021.255. 34934514 PMC8668406

[5] Shu Y, McCauley J. 2017. GISAID: global initiative on sharing all influenza data – from vision to reality. Eurosurveill 22:30494. doi:10.2807/1560-7917.ES.2017.22.13.30494. PMC5388101 28382917

[6] Bernasconi A, Mari L, Casagrandi R, Ceri S. 2021. Data-driven analysis of amino acid change dynamics timely reveals SARS-CoV-2 variant emergence. Sci Rep 11:21068. doi:10.1038/s41598-021-00496-z. 34702903 PMC8548498

[7] McBroome J, Thornlow B, Hinrichs AS, Kramer A, De Maio N, Goldman N, Haussler D, Corbett-Detig R, Turakhia Y. 2021. A daily-updated database and tools for comprehensive SARS-CoV-2 mutation-annotated trees. Mol Biol Evol 12:5819–5824. doi:10.1093/molbev/msab264. PMC8662617 34469548

[8] Chen C, Nadeau S, Yared M, Voinov P, Xie N, Roemer C, Stadler T. 2022. CoV-spectrum: analysis of globally shared SARS-CoV-2 data to identify and characterize new variants. Bioinformatics 38:1735–1737. doi:10.1093/bioinformatics/btab856. PMC8896605 34954792

[9] Rando HM, MacLean AL, Lee AJ, Lordan R, Ray S, Bansal V, Skelly AN, Sell E, Dziak JJ, Shinholster L, McGowan LD, Guebila MB, Wellhausen N, Knyazev S, Boca SM, Capone S, Qi Y, Park Y, Mai D, Sun Y, Boerckel JD, Brueffer C, Byrd JB, Kamil JP, Wang J, Velazquez R, Szeto GL, Barton JP, Goel RR, Mangul S, Lubiana T, Gitter A, Greene CS, COVID-19 Review Consortium. 2021. Pathogenesis, symptomatology, and transmission of SARS-CoV-2 through analysis of viral genomics and structure. mSystems 6:e00095-21. doi:10.1128/msystems.00095-21. PMC8547481 34698547

[10] Rando HM, Wellhausen N, Ghosh S, Lee AJ, Dattoli AA, Hu F, Byrd JB, Rafizadeh DN, Lordan R, Qi Y, Sun Y, Brueffer C, Field JM, Guebila MB, Jadavji NM, Skelly AN, Ramsundar B, Wang J, Goel RR, Park Y, Boca SM, Gitter A, Greene CS, COVID-19 Review Consortium. 2021. Identification and development of therapeutics for COVID-19. mSystems 6:e00233-21. doi:10.1128/mSystems.00233-21. PMC8562484 34726496

[11] Tao K, Tzou PL, Nouhin J, Gupta RK, de Oliveira T, Kosakovsky PS, Fera D, Shafer RW. 2021. The biological and clinical significance of emerging SARS-CoV-2 variants. Nat Rev Genet 22:1–17. doi:10.1038/s41576-021-00408-x. 34535792 PMC8447121

[12] Plante JA, Mitchell BM, Plante KS, Debbink K, Weaver SC, Menachery VD. 2021. The variant gambit: COVID-19’s next move. Cell Host Microbe 29:508–515. doi:10.1016/j.chom.2021.02.020. 33789086 PMC7919536

[13] Sanjuán R, Nebot MR, Chirico N, Mansky LM, Belshaw R. 2010. Viral mutation rates. J Virol 84:9733–9748. doi:10.1128/JVI.00694-10. 20660197 PMC2937809

[14] Peacock TP, Bauer DLV, Barclay WS. 2021. Putative host origins of RNA insertions in SARS-CoV-2 genomes. https://virological.org/t/putative-host-origins-of-rna-insertions-in-sars-cov-2-genomes/761.

[15] Mohammadi E, Shafiee F, Shahzamani K, Ranjbar MM, Alibakhshi A, Ahangarzadeh S, Beikmohammadi L, Shariati L, Hooshmandi S, Ataei B, Javanmard SH. 2021. Novel and emerging mutations of SARS-CoV-2: biomedical implications. Biomed Pharmacother 139:111599. doi:10.1016/j.biopha.2021.111599. 33915502 PMC8062574

[16] Pyrc K, Dijkman R, Deng L, Jebbink MF, Ross HA, Berkhout B, van der Hoek L. 2006. Mosaic structure of human coronavirus NL63, one thousand years of evolution. J Mol Biol 364:964–973. doi:10.1016/j.jmb.2006.09.074. 17054987 PMC7094706

[17] Vankadari N. 2020. Overwhelming mutations or SNPs of SARS-CoV-2: a point of caution. Gene 752:144792. doi:10.1016/j.gene.2020.144792. 32445924 PMC7239005

[18] van Dorp L, Acman M, Richard D, Shaw LP, Ford CE, Ormond L, Owen CJ, Pang J, Tan CCS, Boshier FAT, Ortiz AT, Balloux F. 2020. Emergence of genomic diversity and recurrent mutations in SARS-CoV-2. Infect Genet Evol 83:104351. doi:10.1016/j.meegid.2020.104351. 32387564 PMC7199730

[19] Van Egeren D, Novokhodko A, Stoddard M, Tran U, Zetter B, Rogers M, Pentelute BL, Carlson JM, Hixon M, Joseph-McCarthy D, Chakravarty A. 2021. Risk of rapid evolutionary escape from biomedical interventions targeting SARS-CoV-2 spike protein. PLoS One 16:e0250780. doi:10.1371/journal.pone.0250780. 33909660 PMC8081162

[20] Davies NG, Abbott S, Barnard RC, Jarvis CI, Kucharski AJ, Munday JD, Pearson CAB, Russell TW, Tully DC, Washburne AD, Wenseleers T, Gimma A, Waites W, Wong KLM, van Zandvoort K, Silverman JD, Diaz-Ordaz K, Keogh R, Eggo RM, Funk S, Jit M, Atkins KE, Edmunds WJ, CMMID COVID-19 Working Group, COVID-19 Genomics UK (COG-UK) Consortium. 2021. Estimated transmissibility and impact of SARS-CoV-2 lineage B.1.1.7 in England. Science 372:eabg3055. doi:10.1126/science.abg3055. 33658326 PMC8128288

[21] Ren L, Zhang Y, Li J, Xiao Y, Zhang J, Wang Y, Chen L, Paranhos-Baccalà G, Wang J. 2015. Genetic drift of human coronavirus OC43 spike gene during adaptive evolution. Sci Rep 5:11451. doi:10.1038/srep11451. 26099036 PMC4476415

[22] Cotten M, Robertson DL, Phan MVT. 2021. Unique protein features of SARS-CoV-2 relative to other sarbecoviruses. Virus Evol 7:veab067. doi:10.1093/ve/veab067. 34527286 PMC8385934

[23] Hadfield J, Megill C, Bell SM, Huddleston J, Potter B, Callender C, Sagulenko P, Bedford T, Neher RA. 2018. Nextstrain: real-time tracking of pathogen evolution. Bioinformatics 34:4121–4123. doi:10.1093/bioinformatics/bty407. 29790939 PMC6247931

[24] Martin DP, Weaver S, Tegally H, San JE, Shank SD, Wilkinson E, Lucaci AG, Giandhari J, Naidoo S, Pillay Y, Singh L, Lessells RJ, Gupta RK, Wertheim JO, Nekturenko A, Murrell B, Harkins GW, Lemey P, MacLean OA, Robertson DL, de Oliveira T, Kosakovsky Pond SL, COVID-19 Genomics UK (COG-UK). 2021. The emergence and ongoing convergent evolution of the SARS-CoV-2 N501Y lineages. Cell 184:5189–5200. doi:10.1016/j.cell.2021.09.003. 34537136 PMC8421097

[25] Rambaut A, Holmes EC, O'Toole Á, Hill V, McCrone JT, Ruis C, Du Plessis L, Pybus OG. 2020. A dynamic nomenclature proposal for SARS-CoV-2 lineages to assist genomic epidemiology. Nat Microbiol 5:1403–1407. doi:10.1038/s41564-020-0770-5. 32669681 PMC7610519

[26] O'Toole Á, Scher E, Underwood A, Jackson B, Hill V, McCrone JT, Colquhoun R, Ruis C, Abu-Dahab K, Taylor B, Yeats C, Du Plessis L, Maloney D, Medd N, Attwood SW, Aanensen DM, Holmes EC, Pybus OG, Rambaut A. 2021. Assignment of epidemiological lineages in an emerging pandemic using the pangolin tool. Virus Evol 7:veab064. doi:10.1093/ve/veab064. 34527285 PMC8344591

[27] Parums DV. 2021. Editorial: revised World Health Organization (WHO) terminology for variants of concern and variants of interest of SARS-CoV-2. Med Sci Monit 27:e933622. doi:10.12659/MSM.933622. 34149046 PMC8230247

[28] Planas D, Veyer D, Baidaliuk A, Staropoli I, Guivel-Benhassine F, Rajah MM, Planchais C, Porrot F, Robillard N, Puech J, Prot M, Gallais F, Gantner P, Velay A, Le Guen J, Kassis-Chikhani N, Edriss D, Belec L, Seve A, Courtellemont L, Péré H, Hocqueloux L, Fafi-Kremer S, Prazuck T, Mouquet H, Bruel T, Simon-Lorière E, Rey FA, Schwartz O. 2021. Reduced sensitivity of SARS-CoV-2 variant Delta to antibody neutralization. Nature 596:276–280. doi:10.1038/s41586-021-03777-9. 34237773

[29] Tasakis RN, Samaras G, Jamison A, Lee M, Paulus A, Whitehouse G, Verkoczy L, Papavasiliou FN, Diaz M. 2021. SARS-CoV-2 variant evolution in the United States: high accumulation of viral mutations over time likely through serial founder events and mutational bursts. PLoS One 16:e0255169. doi:10.1371/journal.pone.0255169. 34297786 PMC8301627

[30] Colson P, Levasseur A, Delerce J, Pinault L, Dudouet P, Devaux C, Fournier P-E, La Scola B, Lagier J-C, Raoult D. 2021. Spreading of a new SARS-CoV-2 N501Y spike variant in a new lineage. Clin Microbiol Infect 27:1352.e1–1352.e5. doi:10.1016/j.cmi.2021.05.006. 33991677 PMC8114812

[31] Lucas C, Vogels CBF, Yildirim I, Rothman JE, Lu P, Monteiro V, Gehlhausen JR, Campbell M, Silva J, Tabachnikova A, Peña-Hernandez MA, Muenker MC, Breban MI, Fauver JR, Mohanty S, Huang J, Pearson C, Muyombwe A, Downing R, Razeq J, Petrone M, Ott I, Watkins A, Kalinich C, Alpert T, Brito A, Earnest R, Murphy S, Neal C, Laszlo E, Altajar A, Tikhonova I, Castaldi C, Mane S, Bilguvar K, Kerantzas N, Ferguson D, Schulz W, Landry M, Peaper D, Shaw AC, Ko AI, Omer SB, Grubaugh ND, Iwasaki A, Yale SARS-CoV-2 Genomic Surveillance Initiative. 2021. Impact of circulating SARS-CoV-2 variants on mRNA vaccine-induced immunity. Nature 600:523–529. doi:10.1038/s41586-021-04085-y. 34634791 PMC9348899

[32] Obermeyer F, Schaffner SF, Jankowiak M, Barkas N, Pyle JD, Park DJ, MacInnis BL, Luban J, Sabeti PC, Lemieux JE. 2021. Analysis of 2.1 million SARS-CoV-2 genomes identifies mutations associated with transmissibility. medRxiv doi:10.1101/2021.09.07.21263228. PMC9161372 35608456

[33] Baj A, Novazzi F, Drago Ferrante F, Genoni A, Tettamanzi E, Catanoso G, Dalla Gasperina D, Dentali F, Focosi D, Maggi F. 2021. Spike protein evolution in the SARS-CoV-2 Delta variant of concern: a case series from Northern Lombardy. Emerg Microbes Infect 10:2010–2015. doi:10.1080/22221751.2021.1994356. 34651569 PMC8567936

[34] Baj A, Novazzi F, Pasciuta R, Genoni A, Ferrante FD, Valli M, Partenope M, Tripiciano R, Ciserchia A, Catanoso G, Focosi D, Maggi F. 2021. Breakthrough infections of E484K-harboring SARS-CoV-2 Delta variant, Lombardy, Italy. Emerg Infect Dis 27:3180–3182. doi:10.3201/eid2712.211792. 34499599 PMC8632179

[35] Zhao Z, Sokhansanj BA, Malhotra C, Zheng K, Rosen GL. 2020. Genetic grouping of SARS-CoV-2 coronavirus sequences using informative subtype markers for pandemic spread visualization. PLoS Comput Biol 16:e1008269. doi:10.1371/journal.pcbi.1008269. 32941419 PMC7523987

[36] Qin L, Ding X, Li Y, Chen Q, Meng J, Jiang T. 2021. Co-mutation modules capture the evolution and transmission patterns of SARS-CoV-2. Briefings Bioinformatics 22:bbab222. doi:10.1093/bib/bbab222. 34121111

[37] Pardo-Seco J, Gómez-Carballa A, Bello X, Martinón-Torres F, Salas A. 2021. Pitfalls of barcodes in the study of worldwide SARS-CoV-2 variation and phylodynamics. Zool Res 42:87–93. doi:10.24272/j.issn.2095-8137.2020.364. 33410308 PMC7840454

[38] Nussenblatt V, Roder AE, Das S, de Wit E, Youn JH, Banakis S, Mushegian A, Mederos C, Wang W, Chung M, Pérez-Pérez L, Palmore T, Brudno JN, Kochenderfer JN, Ghedin E. 2021. Year-long COVID-19 infection reveals within-host evolution of SARS-CoV-2 in a patient with B cell depletion. medRxiv doi:10.1101/2021.10.02.21264267. PMC8755281 34940844

[39] Voloch CM, da Silva Francisco R, de Almeida LGP, Brustolini OJ, Cardoso CC, Gerber AL, Guimarães APDC, Leitão IDC, Mariani D, Ota VA, Lima CX, Teixeira MM, Dias ACF, Galliez RM, Faffe DS, Pôrto LC, Aguiar RS, Castiñeira TMPP, Ferreira OC, Tanuri A, de Vasconcelos ATR. 2021. Intra-host evolution during SARS-CoV-2 prolonged infection. Virus Evol 7:veab078. doi:10.1093/ve/veab078. 34642605 PMC8500031

[40] Chen L, Zody MC, Di Germanio C, Martinelli R, Mediavilla JR, Cunningham MH, Composto K, Chow KF, Kordalewska M, Corvelo A, Oschwald DM, Fennessey S, Zetkulic M, Dar S, Kramer Y, Mathema B, Germer S, Stone M, Simmons G, Busch MP, Maniatis T, Perlin DS, Kreiswirth BN. 2021. Emergence of multiple SARS-CoV-2 antibody escape variants in an immunocompromised host undergoing convalescent plasma treatment. mSphere 6:e00480-21. doi:10.1128/mSphere.00480-21. PMC8386433 34431691

[41] Wei C, Shan KJ, Wang W, Zhang S, Huan Q, Qian W. 2021. Evidence for a mouse origin of the SARS-CoV-2 Omicron variant. J Genet Genom 48:1111–1121. doi:10.1016/j.jgg.2021.12.003. PMC8702434 34954396

[42] Kupperschmidt K. 2021. Where did ‘weird’ Omicron come from? Science. 374:1179. doi:10.1126/science.acx9738. 34855502

[43] Rosen G, Garbarine E, Caseiro D, Polikar R, Sokhansanj B. 2008. Metagenome fragment classification using N-mer frequency profiles. Adv Bioinformatics 2008:205969. doi:10.1155/2008/205969. 19956701 PMC2777009

[44] Libbrecht MW, Noble WS. 2015. Machine learning applications in genetics and genomics. Nat Rev Genet 16:321–332. doi:10.1038/nrg3920. 25948244 PMC5204302

[45] Vervier K, Mahé P, Tournoud M, Veyrieras JB, Vert JP. 2016. Large-scale machine learning for metagenomics sequence classification. Bioinformatics 32:1023–1032. doi:10.1093/bioinformatics/btv683. 26589281 PMC4896366

[46] I W, G P, Sc T. 2016. Correct machine learning on protein sequences: a peer-reviewing perspective. Briefings Bioinformatics 17:831–840. doi:10.1093/bib/bbv082. 26411473

[47] Bzhalava Z, Tampuu A, Bała P, Vicente R, Dillner J. 2018. Machine learning for detection of viral sequences in human metagenomic datasets. BMC Bioinformatics 19:336. doi:10.1186/s12859-018-2340-x. 30249176 PMC6154907

[48] Alam MNU, Chowdhury UF. 2020. Short k-mer abundance profiles yield robust machine learning features and accurate classifiers for RNA viruses. PLoS One 15:e0239381. doi:10.1371/journal.pone.0239381. 32946529 PMC7500682

[49] Zhao Z, Yang W, Zhai Y, Liang Y, Zhao Y. 2022. Identify DNA-binding proteins through the extreme gradient boosting algorithm. Front Genet 12:821996. doi:10.3389/fgene.2021.821996. 35154264 PMC8837382

[50] Lau BT, Pavlichin D, Hooker AC, Almeda A, Shin G, Chen J, Sahoo MK, Huang CH, Pinsky BA, Lee HJ, Ji HP. 2021. Profiling SARS-CoV-2 mutation fingerprints that range from the viral pangenome to individual infection quasispecies. Genome Med 13:62. doi:10.1186/s13073-021-00882-2. 33875001 PMC8054698

[51] Basu S, Campbell RH. 2021. Classifying COVID-19 variants based on genetic sequences using deep learning models. bioRxiv doi:10.1101/2021.06.29.450335.

[52] Ali S, Sahoo B, Ullah N, Zelikovskiy A, Patterson M, Khan I. 2021. A k-mer based approach for SARS-CoV-2 variant identification, p 153–164. *In* Wei Y, Li M, Skums P, Cai Z (ed), Bioinformatics research and applications lecture notes in computer science, Springer International Publishing, New York, NY.

[53] Saha I, Ghosh N, Maity D, Seal A, Plewczynski D. 2021. COVID-DeepPredictor: recurrent neural network to predict SARS-CoV-2 and other pathogenic viruses. Front Genet 12:569120. doi:10.3389/fgene.2021.569120. 33643375 PMC7906283

[54] Lemoine F, Blassel L, Voznica J, Gascuel O. 2021. COVID-align: accurate online alignment of hCoV-19 genomes using a profile HMM. Bioinformatics 37:1761–1762. doi:10.1093/bioinformatics/btaa871. 33045068 PMC7745650

[55] Oliveira LS, Gruber A. 2021. Rational design of profile hidden Markov models for viral classification and discovery. *In* Helder IN (ed), Bioinformatics. Exon Publications, Brisbane, Australia. 33877768

[56] LeCun Y, Bengio Y, Hinton G. 2015. Deep learning. Nature 521:436–444. doi:10.1038/nature14539. 26017442

[57] Zou J, Huss M, Abid A, Mohammadi P, Torkamani A, Telenti A. 2019. A primer on deep learning in genomics. Nat Genet 51:12–18. doi:10.1038/s41588-018-0295-5. 30478442 PMC11180539

[58] Schmidt B, Hildebrandt A. 2021. Deep learning in next-generation sequencing. Drug Discov Today 26:173–180. doi:10.1016/j.drudis.2020.10.002. 33059075 PMC7550123

[59] Villegas-Morcillo A, Makrodimitris S, van Ham RCHJ, Gomez AM, Sanchez V, Reinders MJT. 2021. Unsupervised protein embeddings outperform hand-crafted sequence and structure features at predicting molecular function. Bioinformatics 37:162–170. doi:10.1093/bioinformatics/btaa701. 32797179 PMC8055213

[60] Eraslan G, Avsec Ž, Gagneur J, Theis FJ. 2019. Deep learning: new computational modelling techniques for genomics. Nat Rev Genet 20:389–403. doi:10.1038/s41576-019-0122-6. 30971806

[61] Iuchi H, Matsutani T, Yamada K, Iwano N, Sumi S, Hosoda S, Zhao S, Fukunaga T, Hamada M. 2021. Representation learning applications in biological sequence analysis. Comput Struct Biotechnol J 19:3198–3208. doi:10.1016/j.csbj.2021.05.039. 34141139 PMC8190442

[62] Littmann M, Heinzinger M, Dallago C, Weissenow K, Rost B. 2021. Protein embeddings and deep learning predict binding residues for various ligand classes. Sci Rep 11:23916. doi:10.1038/s41598-021-03431-4. 34903827 PMC8668950

[63] Le NQK, Ho QT. 2021. Deep transformers and convolutional neural network in identifying DNA N6-methyladenine sites in cross-species genomes. Methods. S1046-2023(21)00274-7. doi:10.1016/j.ymeth.2021.12.004. 34915158

[64] Hie B, Zhong ED, Berger B, Bryson B. 2021. Learning the language of viral evolution and escape. Science 371:284–288. doi:10.1126/science.abd7331. 33446556

[65] Devlin J, Chang MW, Lee K, Toutanova K. 2019. BERT: pre-training of deep bidirectional transformers for language understanding, p 4171–4186. *In* Proceedings of the 2019 Conference of the North American Chapter of the Association for Computational Linguistics: Human Language Technologies, Volume 1 (Long and Short Papers). Association for Computational Linguistics, Minneapolis, Minnesota.

[66] Rives A, Meier J, Sercu T, Goyal S, Lin Z, Liu J, Guo D, Ott M, Zitnick CL, Ma J, Fergus R. 2021. Biological structure and function emerge from scaling unsupervised learning to 250 million protein sequences. Proc Natl Acad Sci USA 118:e2016239118. doi:10.1073/pnas.2016239118. 33876751 PMC8053943

[67] Elnaggar A, Heinzinger M, Dallago C, Rehawi G, Wang Y, Jones L, Gibbs T, Feher T, Angerer C, Steinegger M, Bhowmik D, Rost B. 2021. ProtTrans: towards cracking the language of lifes code through self-supervised deep learning and high performance computing. IEEE Trans Pattern Anal Mach Intell doi:10.1109/TPAMI.2021.3095381. 34232869

[68] Brandes N, Ofer D, Peleg Y, Rappoport N, Linial M. 2022. ProteinBERT: a universal deep-learning model of protein sequence and function. Bioinform doi:10.1093/bioinformatics/btac020. PMC9386727 35020807

[69] Mullick B, Magar R, Jhunjhunwala A, Barati FA. 2021. Understanding mutation hotspots for the SARS-CoV-2 spike protein using shannon entropy and K-means clustering. Comput Biol Med 138:104915. doi:10.1016/j.compbiomed.2021.104915. 34655896 PMC8492016

[70] Sokhansanj BA, Zhao Z, Rosen GL. 2021. Interpretable and predictive deep modeling of the SARS-CoV-2 spike protein sequence. medRxiv doi:10.1101/2021.12.26.21268414. PMC9774807 36552295

[71] Beguir K, Skwark MJ, Fu Y, Pierrot T, Carranza NL, Laterre A, Kadri I, Lui BG, Sänger B, Liu Y, Poran A, Muik A, Sahin U. 2021. Early computational detection of potential high risk SARS-CoV-2 variants. bioRxiv doi:10.1101/2021.12.24.474095. PMC9892295 36774893

[72] Fowler DM, Fields S. 2014. Deep mutational scanning: a new style of protein science. Nat Methods 11:801–807. doi:10.1038/nmeth.3027. 25075907 PMC4410700

[73] Gelman S, Fahlberg SA, Heinzelman P, Romero PA, Gitter A. 2021. Neural networks to learn protein sequence–function relationships from deep mutational scanning data. Proc National Acad Sci USA 118:e2104878118. doi:10.1073/pnas.2104878118. PMC8640744 34815338

[74] Chen J, Wang R, Gilby NB, Wei GW. 2021. Omicron (B.1.1.529): infectivity, vaccine breakthrough, and antibody resistance. arXiv 2112.01318v1. 10.1021/acs.jcim.1c01451 PMC8751645 34989238

[75] Linardatos P, Papastefanopoulos V, Kotsiantis SB. 2021. Explainable AI: a review of machine learning interpretability methods. Entropy (Basel) 23:18. doi:10.3390/e23010018. PMC7824368 33375658

[76] Murdoch WJ, Singh C, Kumbier K, Abbasi-Asl R, Yu B. 2019. Definitions, methods, and applications in interpretable machine learning. Proc Natl Acad Sci USA 116:22071–22080. doi:10.1073/pnas.1900654116. 31619572 PMC6825274

[77] Auslander N, Gussow AB, Koonin EV. 2021. Incorporating machine learning into established bioinformatics frameworks. Int J Mol Sci 22:2903. doi:10.3390/ijms22062903. 33809353 PMC8000113

[78] Watson DS. 2021. Interpretable machine learning for genomics. Hum Genet doi:10.1007/s00439-021-02387-9. PMC8527313 34669035

[79] Montavon G, Samek W, Müller KR. 2018. Methods for interpreting and understanding deep neural networks. Digit Signal Process 73:1–15. doi:10.1016/j.dsp.2017.10.011.

[80] Shrikumar A, Greenside P, Kundaje A. 2017. Learning important features through propagating activation differences, p 3145–3153. *In* International Conference on Machine Learning (PMLR).

[81] Simonyan K, Vedaldi A, Zisserman A. 2014. Deep inside convolutional networks: visualising image classification models and saliency maps. arXiv 1312.6034.

[82] Jha A, Aicher JK, Gazzara MR, Singh D, Barash Y. 2020. Enhanced integrated gradients: improving interpretability of deep learning models using splicing codes as a case study. Genome Biol 21:149. doi:10.1186/s13059-020-02055-7. 32560708 PMC7305616

[83] Bahdanau D, Cho K, Bengio Y. 2014. Neural machine translation by jointly learning to align and translate. arXiv 1409.0473.

[84] Xu K, Ba J, Kiros R, Cho K, Courville AC, Salakhutdinov R, Zemel RS, Bengio Y. 2015. Show, attend and tell: neural image caption generation with visual attention. arXiv 1502.03044.

[85] Rush AM, Chopra S, Weston J. 2015. A neural attention model for abstractive sentence summarization. arXiv 1509.00685.

[86] Yang Z, Yang D, Dyer C, He X, Smola A, Hovy E. 2016. Hierarchical attention networks for document classification, p 1480–1489. *In* Proceedings of the 2016 Conference of the North American Chapter of the Association for Computational Linguistics: Human Language Technologies.

[87] Zhou P, Shi W, Tian J, Qi Z, Li B, Hao H, Xu B. 2016. Attention-based bidirectional long short-term memory networks for relation classification, p 207–212. *In* Proceedings of the 54th Annual Meeting of the Association for Computational Linguistics.

[88] Deming L, Targ S, Sauder N, Almeida D, Ye CJ. 2016. Genetic architect: discovering genomic structure with learned neural architectures. arXiv 1605.07156.

[89] Lanchantin J, Singh R, Lin Z, Qi Y. 2016. Deep motif: visualizing genomic sequence classifications. arXiv 1605.01133.

[90] Dey S, Luo H, Fokoue A, Hu J, Zhang P. 2018. Predicting adverse drug reactions through interpretable deep learning framework. BMC Bioinformatics 19:476. doi:10.1186/s12859-018-2544-0. 30591036 PMC6300887

[91] Zhao Z, Woloszynek S, Agbavor F, Mell JC, Sokhansanj BA, Rosen GL. 2021. Learning, visualizing and exploring 16S rRNA structure using an attention-based deep neural network. PLoS Comput Biol 17:e1009345. doi:10.1371/journal.pcbi.1009345. 34550967 PMC8496832

[92] Zhao Z, Rosen G. 2020. Visualizing and annotating protein sequences using a deep neural network, p 506–510. *In* 2020 54th Asilomar Conference on Signals, Systems, and Computers.

[93] Vaswani A, Shazeer N, Parmar N, Uszkoreit J, Jones L, Gomez AN, Kaiser Ł, Polosukhin I. 2017. Attention is all you need, p 6000–6010. *In* Proceedings of the 31st International Conference on Neural Information Processing Systems NIPS’17, Curran Associates Inc., Red Hook, NY, USA.

[94] Vig J. 2019. BertViz: a tool for visualizing multihead self-attention in the BERT model. *In* ICLR Workshop: Debugging Machine Learning Models.

[95] Clauwaert J, Menschaert G, Waegeman W. 2021. Explainability in transformer models for functional genomics. Briefings Bioinform 22:bbab060. doi:10.1093/bib/bbab060. PMC8425421 33834200

[96] Kobayashi G, Kuribayashi T, Yokoi S, Inui K. 2020. Attention is not only a weight: analyzing transformers with vector norms. arXiv 2004.10102 [cs].

[97] Jain S, Wallace BC. 2019. Attention is not explanation. arXiv 1902.10186.

[98] Van Noorden R. 2021. Scientists call for fully open sharing of coronavirus genome data. Nature 590:195–196. doi:10.1038/d41586-021-00305-7. 33542487

[99] Brito AF, Semenova E, Dudas G, Hassler GW, Kalinich CC, Kraemer MUG, Hill SC, Sabino EC, Pybus OG, Dye C, Bhatt S, Flaxamn S, Suchard MA, Grubaugh ND, Baele G, Faria NR, Danish Covid-19 Genome Consortium. 2021. Global disparities in SARS-CoV-2 genomic surveillance. medRxiv doi:10.1101/2021.08.21.21262393.

[100] Spratt DE, Chan T, Waldron L, Speers C, Feng FY, Ogunwobi OO, Osborne JR. 2016. Racial/ethnic disparities in genomic sequencing. JAMA Oncol 2:1070–1074. doi:10.1001/jamaoncol.2016.1854. 27366979 PMC5123755

[101] Schriml LM, Chuvochina M, Davies N, Eloe-Fadrosh EA, Finn RD, Hugenholtz P, Hunter CI, Hurwitz BL, Kyrpides NC, Meyer F, Mizrachi IK, Sansone SA, Sutton G, Tighe S, Walls R. 2020. COVID-19 pandemic reveals the peril of ignoring metadata standards. Sci Data 7:188. doi:10.1038/s41597-020-0524-5. 32561801 PMC7305141

[102] Jacot D, Pillonel T, Greub G, Bertelli C. 2021. Assessment of SARS-CoV-2 genome sequencing: quality criteria and low-frequency variants. J Clin Microbiol 59:e00944-21. doi:10.1128/JCM.00944-21. PMC8451431 34319802

[103] Lagerborg KA, Normandin E, Bauer MR, Adams G, Figueroa K, Loreth C, Gladden-Young A, Shaw BM, Pearlman LR, Berenzy D, Dewey HB, Kales S, Dobbins ST, Shenoy ES, Hooper D, Pierce VM, Zachary KC, Park DJ, MacInnis BL, Tewhey R, Lemieux JE, Sabeti PC, Reilly SK, Siddle KJ. 2022. Synthetic DNA spike-ins (SDSIs) enable sample tracking and detection of inter-sample contamination in SARS-CoV-2 sequencing workflows. Nat Microbiol 7:108–119. doi:10.1038/s41564-021-01019-2. 34907347 PMC8923058

[104] Hasell J, Mathieu E, Beltekian D, Macdonald B, Giattino C, Ortiz-Ospina E, Roser M, Ritchie H. 2020. A cross-country database of COVID-19 testing. Sci Data 7:345. doi:10.1038/s41597-020-00688-8. 33033256 PMC7545176

